# PRMT5 inhibitors: Therapeutic potential in pancreatic cancer

**DOI:** 10.1016/j.tranon.2025.102366

**Published:** 2025-03-28

**Authors:** Carolin Schneider, Valentina Spielmann, Christian J. Braun, Günter Schneider

**Affiliations:** a Department of General, Visceral and Pediatric Surgery, University Medical Center Göttingen, Göttingen, 37075, Germany; b Clinical Research Unit 5002, KFO5002, University Medical Center Göttingen, Göttingen, 37075, Germany; c Department of Pediatrics, Dr. von Hauner Children's Hospital, University Hospital, LMU Munich, Munich, 80337, Germany; d CCC-N (Comprehensive Cancer Center Lower Saxony), Göttingen, 37075, Germany; e Institute for Translational Cancer Research and Experimental Cancer Therapy, Technical University Munich, Munich, 81675, Germany

**Keywords:** Pancreatic cancer, KRAS, PRMT5, Resistance, Immunotherapies

## Abstract

Pancreatic ductal adenocarcinoma (PDAC) is predominantly driven by mutations in the Kirsten rat sarcoma virus (KRAS) oncogene. Due to the pivotal role of KRAS in PDAC pathogenesis, KRAS inhibitors (KRASi) have recently demonstrated initial signs of clinical efficacy. However, considering currently documented response rates and the resistance development to KRASi, additional targeted therapies are needed. In this context, we provide a summary of recent preclinical and clinical findings on protein arginine methyltransferase 5 (PRMT5) inhibitors (PRMT5i) and their implications for PDAC. PRMT5 has been linked to key oncogenic processes, including epithelial-mesenchymal transition (EMT), MYC and Hippo signaling pathways, glycolysis, and therapy resistance. With further advancements and optimization of PRMT5i-based therapies, these inhibitors hold significant potential as therapeutic agents for PDAC treatment. Therefore, we synthesize the current understanding of PRMT5i and highlight promising directions for future developments in PDAC.

## Highlights

• Clinically viable MTA-cooperative PRMT5 inhibitors with a favorable therapeutic window are now available.
• A distinct PRMT5 inhibitor-sensitive PDAC subtype has been identified.
• PRMT5 inhibitor-based combination therapies should be developed as a strategy for the treatment of PDAC.

## Graphical abstract

**Figure:**
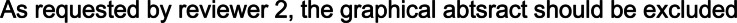

## Introduction

### KRAS inhibitors: therapeutic opportunities in PDAC

The incidence of Pancreatic Ductal Adenocarcinoma (PDAC) continues to rise, with approximately 500,000 new cases diagnosed globally in 2022. The growing incidence of early-onset PDAC along with the inadequate effectiveness of current systemic therapies, raises significant concerns about the future management of this challenging disease. Given that mutated Kirsten rat sarcoma virus (KRAS) oncogene drives the disease in 90 % of cases, it is likely that KRAS inhibitors (KRASi) will soon become a part of standard of clinical care [1,2]. However, current clinical data reveal overall response rates of approximately 30 % of patients receiving KRASi monotherapies, with the frequent development of resistance [1,2]. This underscores the need to identify and implement additional therapeutic targets to improve long-term outcomes. Recent advancements in the development of protein arginine methyltransferases (PRMT) 5 (PRMT5) inhibitors (PRMT5is) represent a significant step forward and a potential therapeutic option for pancreatic cancer treatment.

## Role of PRMT5 in PDAC

**Fig. 1:**
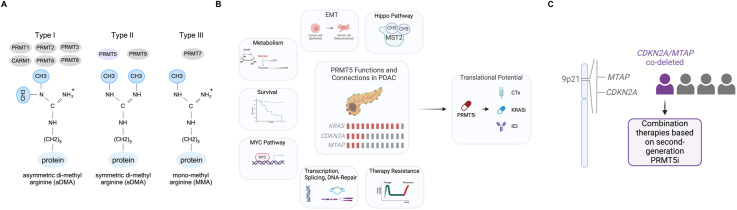
PRMTs and PRMT5 function in PDAC A The nine protein arginine methyltransferases (PRMTs) are classified into three types: Type I enzymes, which catalyze asymmetric demethylation (aDMA); Type II enzymes, which mediate symmetric demethylation (sDMA); and Type III enzymes, which catalyze monomethylation (MMA). All PRMTs have the ability to perform monomethylation of arginine. B The functions and the therapeutic potential of PRMT5 are outlined, with a particular emphasis on its role in PDAC. Additionally, the OncoPrint highlights the PDAC driver gene *KRAS* and the tumor suppressor *CDKN2A*, along with the co-deletion of *MTAP*. Red-box: gene altered, grey-box: not-altered, CTx: chemotherapy, EMT: epithelial to mesenchymal transition; ICI: immune checkpoint inhibitors, KRASi: KRAS inhibitor, MST2: mammalian sterile 20-like kinases 2, PRMT5i: PRMT5 inhibitor. C The human 9p21 locus, which harbors the *Cyclin-dependent Kinase Inhibitor 2A* (*CDKN2A*) and *methylthioadenosine phosphorylase* (*MTAP*) genes, is illustrated. Co-deletion of *CDKN2A* and *MTAP* creates a collateral vulnerability that can be targeted using combination therapies based on second-generation PRMT5is.

Post-translational modifications (PTMs) play a crucial role in regulating protein functions, and disruptions in PTM networks are linked to cancer. Among these, arginine methylation, catalyzed by PRMTs [3], represents a distinct PTM with increasing evidence supporting its crucial role in the pathobiology of different neoplastic diseases. To date, nine PRMTs have been identified in mammals. All PRMTs catalyze arginine mono-methylation; however, PRMT5, a type II protein arginine methyltransferase (Fig. 1A), specifically catalyzes the formation of symmetric dimethylarginine (sDMA) [3]. PRMT5 forms a homotetramer that interacts with the methylosome protein 50 (MEP50/WDR77) to build an active hetero-octameric complex. PRMT5 methylates various proteins, including histones, RNA-binding proteins (RBPs), transcription factors, DNA repair proteins, and proteins involved in signal transduction.

High expression of PRMT5 is associated with a more aggressive PDAC subtype and is linked to shorter survival rates [4]. Furthermore, the methyltransferase has been connected to processes such as epithelial-mesenchymal transition (EMT), the myelocytomatosis oncogene (MYC), Hippo signaling, and glycolysis (Fig. 1B) [[4], [5], [6], [7], [8]]. Notably, a PRMT5i-sensitive subtype of PDAC has been consistently identified [[4], [5], [6], [7],^9^], and PRMT5is have been shown to enhance the efficacy of gemcitabine [10], poly-ADP ribose polymerase (PARP) inhibitors [11], or transforming growth factor beta (TGF-β) signaling inhibitors [12].

### Development of advanced PRMT5 inhibitors

While these findings support the advancement of PRMT5is into clinical testing for PDAC, the results from Phase I clinical trials of first-generation PRMT5 inhibitors have tempered these efforts. First-generation inhibitors, as illustrated in Table 1 for selected PRMT5is, have been shown to possess a narrow therapeutic index, likely due to their on-target activity in rapidly proliferating cells, such as those in the bone marrow or gastrointestinal tract. Like other methyltransferases, PRMT5 utilizes S-Adenosyl methionine (SAM) as a methyl group donor. The methyltransferase domain of PRMT5 features two binding pockets: one for SAM and the other for the substrate. Interestingly, genetic screens demonstrated that deficiency in the methylthioadenosine phosphorylase (*MTAP*) gene, an enzyme in the methionine salvage pathway, generates a dependency on PRMT5 [[13], [14], [15]]. The *MTAP* gene is located on chromosome 9p21, near the *Cyclin-dependent Kinase Inhibitor 2A* (*CDKN2A*) tumor suppressor gene (Fig. 1C). Co-deletion of *CDKN2A* and *MTAP* occurs in approximately 26 % of PDAC cases (Fig. 1B) [14]. Loss of MTAP results in the accumulation of methylthioadenosine (MTA) in cancer cells, that partially inhibits the PRMT5 methyltransferase activity. The first generation of PRMT5i were substrate-competitive and SAM-cooperative (*e.*g*.* GSK3326595) or SAM-competitive (*e.*g*.* JNJ64619178, PF06939999, LLY283) and showed no selectivity for *MTAP*-deleted cancers. This contrasts with the MTA-cooperative PRMT5 inhibitors, which were developed in the recent years and include MRTX1719 [16], AM-9747, a lead for AMG193 [17], TNG908 [18], TNG462 [19], AZD3470 [20], or AZ-PRMT5i-1 [21].

**Table 1:** Preclinical and clinical activity of selected PRMT5is [[16], [17], [22], [23],[38], [39], [40], [41], [42], [43]].

Abbreviations - DCR: disease control rate, ORR: overall response rate, PD: progressive disease, PR: partial remission, SD: stable disease, TGI: tumor growth inhibition, TRAE: treatment-related adverse events.

MRTX1719 was discovered by structure-based drug design of a lead, defined by a fragment-based screen [16]. The inhibitor demonstrates selective activity against *MTAP*-depleted cancers both *in vitro* and *in vivo*, effectively reducing sDMA levels in cancer cells while sparing normal tissues [22], thereby showcasing a favorable therapeutic window. MRTX1719 is particularly potent in *MTAP*-deleted PDAC cell lines and exhibits significant *in vivo* efficacy in PDAC patient-derived Xenotransplant (PdX) models (Table 1) [22]. Treatment with MRTX1719 resulted in the reduction of sDMA in spliceosomal, transcriptional, and cell cycle-related proteins. In line with the critical role of sDMA in spliceosome function, MRTX1719 treatment also led to an increased expression of transcripts containing detained introns [22]. Initial clinical data indicate a therapeutic window with no dose-limiting toxicities observed at the investigated dose levels [22]. Additionally, clinical partial responses have been noted in cancers with *CDKN2A*/*MTAP* co-deletions.

An alternative approach utilized a DNA-encoded library to screen for MTA-cooperative inhibitors, resulting in the discovery of an aminoquinoline compound with PRMT5 inhibitory activity. Through lead optimization, the clinical candidate PRMT5i, AMG193, was developed and revealed a 60-fold higher potency to bind PRMT5 in the presence of MTA [17]. Similarly, selectivity for *MTAP*-deleted cancer cell lines was observed, with sensitivity to AMG193 correlated with a genetic dependency on PRMT5 [17]. Mechanistically, AMG193 demonstrated a strong impact on the regulation of alternative splicing, resulting in cell cycle arrest and induction of DNA damage. Furthermore, PDAC PdX models responded to AMG193 monotherapy [17]. The recently published Phase I clinical data for AMG193 demonstrate a favorable safety profile. As anticipated for a PRMT5i that cooperates with MTA, no dose-limiting myelosuppression was observed (Table 1) [23]. Among the patients with PDAC treated with an active dose of AMG193, one partial remission was observed [23]. Additionally, AMG193 is currently being investigated in combination with chemotherapy for PDAC (NCT06360354).

### The future of PRMT5is in PDAC - Combination Therapies

Given the therapeutic window of MTA-cooperative PRMT5is, the significant fraction of *CDKN2A/MTAP* co-deleted PDACs, and the supporting preclinical and clinical data, advancing PRMT5is as a potential treatment option for PDAC is a compelling strategy. A critical next step will be to identify effective combination therapies and map PRMT5 resistance trajectories to enhance clinical response rates and extend progression-free survival in PRMT5i-based treatments. Screening experiments in Paclitaxel-resistant triple-negative breast cancers revealed a particular sensitivity of chemotherapy resistant cells towards PRMT5is [24]. Consistently, a recent study has shown that PRMT5is can reverse Gemcitabine resistance in PDAC cells [8]. These findings indicate a broader dependence of therapy-resistant cells on PRMT5 and additionally support the rationale for testing PRMT5is in combination with chemotherapy, as investigated in the ongoing AMG193 PDAC trial (NCT06360354).

Another interesting approach is to combine MTA-cooperative PRMT5is with KRASi. The advantages of combining AMG193 with the KRAS^G12C^ inhibitor Sotorasib have already been documented in MTAP-deleted MiaPaCa2 cell *in vitro* and in xenotransplant models [17]. Sotorasib typically induces a G1-phase arrest in PDAC models [25], an effect that is further enhanced by co-treatment with AMG193 [17]. Given the low frequency of KRAS^G12C^ mutations of only 1 % in PDAC, it is important to highlight that PRMT5is may synergize with other KRASi (following data published as abstracts). In the KP4 cell line PDAC models, the combination of the KRAS^G12D^ inhibitor MRTX1133 was reported to induce synergistic tumor regression with MRTX1719 [26]. Consistent with the increased G1 phase arrest observed in the combination of AMG193 and Sotorasib [17], MRTX1133 and MRTX1719 have been reported to converge on the activation of the tumor suppressor retinoblastoma (RB) protein [26]. Furthermore, MRTX1719 has been described to sensitize PDAC cells to the KRAS^G12C^ inhibitor MRTX849 and the KRAS^G12D^ inhibitor MRTX1133 [27]. To further advance these therapeutic concepts, it will be important to decipher the molecular underpinning of the synergism. Given the significant role of Yes-associated Protein 1 (YAP1) / transcriptional coactivator with PDZ-binding motif (TAZ) in resistance to KRAS^G12C^ inhibitors [2,28], it may be valuable to investigate whether PRMT5′s impact on the Hippo signaling pathway contributes to the observed synergy between KRAS and PRMT5 inhibitors [7].

### Immunotherapeutic aspects of PRMT5 inhibition

Importantly, in PDAC models, KRASi also act *in vivo* by remodeling the tumor microenvironment (TME), with alteration of cancer-associated fibroblasts, macrophages, and T-cells. It was demonstrated that CD8+ *T*-cells augment the antitumor activity of KRASi by killing tumor cells via the FAS-FASL system [29,30]. Thus, combining PRMT5i with KRASi may present an intriguing immunotherapeutic approach, as it could potentially enhance immune system engagement and improve therapeutic outcomes. Firstly, splicing modulators have been shown to generate splicing-derived neoantigens that can be presented on major histocompatibility complex (MHC) class I molecules capable of triggering CD8+ *T* cell responses [31]. Secondly, in melanoma models, knockdown of PRMT5 has been shown to reduce tumor growth in immunocompetent mice, but not in immunodeficient ones. Mechanistically, PRMT5 methylates IFN-γ–inducible protein 16 (IFI16), a component of the cyclic GMP-AMP synthase (cGAS)/ stimulator of interferon genes (STING) pathway. The methylation of IFI16 by PRMT5 reduced double stranded DNA-mediated interferon and chemokine production. Additionally, PRMT5 represses the transcription of nucleotide-binding oligomerization domain-like receptor family caspase recruitment domain containing 5 (NLRC5), a transcription factor that promotes the expression of genes related to MHC class I antigen presentation [32]. Therefore, increased interferon and chemokine expression and presentation of antigens via MHC class I upon PRMT5 inhibition sensitizes the investigated melanoma models to immune checkpoint therapy [32]. Notably, the connections between PRMT5 and the interferon pathway are context-dependent. For instance, DNA replication stress increases PRMT5 nuclear localization and sDMA levels, both of which are necessary for inducing interferon-stimulated genes [33]. Additionally, MTA accumulation in *MTAP*-deleted cancers may influence the TME [34], and PRMT5 has a significant role in T-cell biology [35]. The context dependency of PRMT5 functions and a role of PRMT5 in immune cells could pose challenges for incorporating immune-assisted eradication strategies into PRMT5i-based therapies. However, recent data show that MRTX1719 treatment induces minimal immunosuppression [36]. Furthermore, MRTX1719 sensitizes *MTAP*-deleted tumors to T-cell-mediated killing, explaining the effectiveness of combining MRTX1719 with anti-PD1 therapy observed *in vivo* models [36]. To fully understand the therapeutic potential of PRMT5i in combination with immunotherapies, further research is needed to clarify the effects of PRMT5 inhibition on the TME. Deciphering the molecular mechanisms behind any potential synergistic effects with immunotherapy will be essential for optimizing combined treatment strategies.

## Conclusions

Despite over 1000 clinical trials were conducted for metastatic PDAC since 2000 [37], improvements in patient prognosis remain disappointing. However, we are now entering an exciting new era with promising clinical indications for the efficacy of targeted therapies. While further work is needed to develop effective PRMT5i containing combination therapies, the existing data discussed here highlight the promising potential of advancing MTA-cooperative PRMT5is for PDAC treatment.

## CRediT authorship contribution statement

**Carolin Schneider:** Writing – review & editing, Writing – original draft, Formal analysis, Conceptualization. **Valentina Spielmann:** Writing – review & editing, Writing – original draft, Formal analysis, Conceptualization. **Christian J. Braun:** Writing – review & editing, Formal analysis, Conceptualization. **Günter Schneider:** Writing – review & editing, Writing – original draft, Formal analysis, Conceptualization.

## Declaration of competing interest

The authors declare that they have no known competing financial interests or personal relationships that could have appeared to influence the work reported in this paper.

The author GS is an Co-Editor-in-Chief for this journal and was not involved in the editorial review or the decision to publish this article.
